# A mixed methods study of the impact of consultant overnight working in an English Emergency Department

**DOI:** 10.1136/emermed-2018-207571

**Published:** 2018-08-09

**Authors:** Marion L Penn, Thomas Monks, Catherine Pope, Mike Clancy

**Affiliations:** 1 NIHR CLAHRC Wessex, University of Southampton Faculty of Health Sciences, Southampton, UK; 2 Emergency Department, University Hospital Southampton NHS Foundation Trust, Southampton, UK

**Keywords:** emergency department, efficiency, safety, quality

## Abstract

**Background:**

There is a growing expectation that consultant-level doctors should be present within an ED overnight. However, there is a lack of robust evidence substantiating the impact on patient waiting times, safety or the workforce.

**Objectives:**

To evaluate the impact of consultant-level doctors overnight working in ED in a large university hospital.

**Methods:**

We conducted a controlled interrupted time series analysis to study ED waiting times before and after the introduction of consultant night working. Adverse event reports (AER) were used as a surrogate for patient safety. We conducted interviews with medical and nursing staff to explore attitudes to night work.

**Results:**

The reduction seen in average time in department relative to the day, following the introduction of consultant was non-significant (−12 min; 95% CI −28 to 4, p=0.148). Analysis of hourly arrivals and departures indicated that overnight work was inherited from the day. There were three (0.9%) moderate and 0 severe AERs in 1 year. The workforce reported that night working had a negative impact on sleep patterns, performance and well-being and there were mixed views about the benefits of consultant night presence. Additional time off during the day acted as compensation for night work but resulted in reduced contact with ED teams.

**Conclusions:**

Our single-site study was unable to demonstrate a clinically important impact of consultant night working on total time patients spend in the department. Our analysis suggests there may be more potential to reduce total time in department during the day, at our study site. Negative impacts on well-being, and likely resistance to consultant night working should not be ignored. Further studies of night working are recommended to substantiate our results.

**Key messages**

**What is already known on this subject**

- Evidence about the benefits and disbenefits of ED consultant overnight working is limited and conflicting. In a single study, consultants self-report a negative impact on job satisfaction, early retirement and fatigue. A general review of shift work found an association with an increased risk of vascular events. Two small sample pre-2010 case reports from the UK found potential benefits of shorter waiting times and reduced admissions.

**What this study adds**

- In our study setting, we were unable to demonstrate a clinically important impact of consultant night working on patient total time in department or adverse events. Staff report a positive impact on junior doctor and nursing workforce job satisfaction. Our results suggest that overnight ED waiting time performance issues are potentially rooted in process problems occurring during the day and early evening. Before widespread adoption of consultant night working in EDs in the UK is considered, further studies exploring a wider range of outcomes must be conducted.

## Introduction

Most EDs in the UK do not have consultant-level doctors present beyond midnight. Instead they are available on call. In recent years the UK’s 7-day hospital agenda^1^ and waiting time performance^2^ have prompted calls for rostering consultant-level doctors overnight.

The rationale for 24 hours’ consultant presence is threefold. First, sick patients continue to arrive at night (and might be sicker) and require the same expertise as patients who arrive during the day. Second, ED staff welcome senior clinical decision-making and managerial support. Third, consultant presence may have a positive impact on patient waiting times^3 4^—a key performance metric within UK hospitals.

However, there is also the potential for negative effects. A longitudinal study in the USA suggested that night working was associated with reduced job satisfaction, fatigue and poor sleep patterns,^5^ and was a factor in decisions to retire. Shift work is associated with an increased risk of vascular events indicating potential harm to staff working such shifts.^6^

Evidence regarding overnight working in ED is derived mainly from two small studies.^3 4^ Given the cost implications of 24 hours’ consultant presence, more evidence is needed. This study reports a mixed methods analysis of consultant night working at a large university hospital in the UK.

Prior to commencing our study we identified four English EDs who had introduced 7-day night working and conducted a brief telephone interview with the clinical director in each to understand the reasons for adopting this night working pattern and details about remuneration. Reasons given included organisational and consultant concerns about staffing, performance and leadership. The numbers of full-time consultants needed to populate the rotas ranged from 15 to 25, with a frequency of nights worked ranging from 7 in 15 weeks to 7 in 22 weeks. Remuneration ranged from twice to four times (when consultants worked without middle grade doctors) that of the office hours rate.

## Objectives

We sought to answer three questions:

- Do nights with a rostered consultant presence have a lower total time in ED for patients?
- Are nights with a rostered consultant presence in the ED safer?
- What are the views of the clinical workforce on overnight working?

## Methods

The study used a mixed methods design to investigate overnight working in the ED, analysing routine data and interviewing staff.

### Study setting

The study took place at a large English university hospital with a designated major trauma, stroke and cardiac centre. The ED employs 22 consultant doctors, present 08:00–24:00 on weekdays, overnight Monday to Thursday and on call after 24:00 Friday to Sunday. There is one specialist registrar year 3 (ST3) and one specialist registrar year 4 (ST4) or above overnight Monday to Thursday, and two ST4’s or above Friday to Sunday. Seven junior doctors (below ST3 grade) work between 18:00 and 03:00 and three from 22:00 to 08:00. The department began rostering a single consultant to be present overnight on a Monday to Thursday from November 2015.

### Quantitative methods

#### Data sources

We analysed routinely collected data January 2013 to June 2017 on the number of arrivals, time to be seen and time to departure, of all adults attending the ED. Overnight was defined as between 22:00 and 06:00 the following day.

Adverse event reports (AER) are completed voluntarily by staff and are classified from near miss to severe impact and we used this a surrogate measure of safety. Using the AER database all reports classified as severe or moderate generated on the nights where consultants consistently worked overnight (Monday to Thursday) were compared with when they were on call (Friday to Sunday) for the period June 2016 to July 2017.

#### Study variables

We hypothesised that the introduction of a consultant overnight would give an abrupt and permanent shift in the *total time in the department* experienced by a patient who arrived during 22:00–06:00 period. Over the 24-hour day, we explored the *minutes to be first seen* by a doctor following triage and the hourly *occupancy change*: the average difference between the number of hourly arrivals to the department and the number departing. Lastly, we used the frequency of *AERs* as a surrogate measure of safety.

#### Statistical analysis

We conducted a retrospective analysis of total time in department using a controlled interrupted time series (ITS) design to account for biases in the level and trend at baseline.^7 8^ We used segmented regression^9^ with adjustments for seasonality as well as autocorrelation in the residuals. Full modelling details and a detailed explanation of ITS are available in the online supplementary appendix. The data were divided into two segments of monthly intervals representing before and after the introduction of consultant night-time presence: (1) January 2013 to October 2015 (n=34) and (2) November 2015 to June 2017 (n=20). Each data point represents the average total time in department for a month. We used 06:00–22:00 hours performance as a quasicontrol group to adjust for intrahospital effects that might influence ED such as improved ‘patient flow’ allowing more timely emergency admissions.

10.1136/emermed-2018-207571.supp1 Supplementary data

We interviewed senior clinicians to identify any interventions that took place close to November 2015. Friday to Sunday were excluded as a control group, due to an increase in middle grade staffing during 2016. There were no simultaneous ED interventions on a Monday to Thursday reported.

### Qualitative methods

We conducted semistructured interviews with senior staff working in the study setting, focusing particularly on the consultant staff affected by proposed changes to night working. Interviewees were purposively sampled to include staff currently working nights and those who did not, and to ensure a range of sociodemographic characteristics and those with/without caring responsibilities. Staff were informed of the research through team meetings and invited in person by the researcher to be interviewed. One interview was by telephone, two were group interviews. The remaining face-to-face interviews took place in a private room in the ED. Handwritten notes were made and subsequently transcribed and anonymised.

The interviews were conducted by an experienced, non-clinical researcher (CP) and explored (1) current work patterns, (2) views about night working by consultant staff, and (3) the impact of night working.

All those approached to be interviewed agreed. Interviewees included 11 consultant grade doctors and four middle grade doctors. Two senior nurses were also interviewed to explore themes identified by middle grade doctors about their preferences for consultant cover at night. Given the small numbers involved and the possibility of attribution the quotes presented below only identify consultant and non-consultant status.

The analysis of the interview data was descriptive and began with transcription of handwritten notes immediately after the interview, followed by reading, open coding and identification of broad themes. Emerging interpretations were checked by conferring with the team and/or asking subsequent interviewees for additional detail.

## Results

### Quantitative results

#### Total time in department

In the 54 months studied there were 186 500 adult Monday to Thursday ED attendances. A total of 43 105 (23.1%) of these were between the hours of 22:00 and 06:00. There were 26 780 (62.1%) adult attendances overnight in the 34 months prior to the introduction of a consultant overnight and 16 325 (37.9%) in the 20 months afterwards.

**Figure 1:**
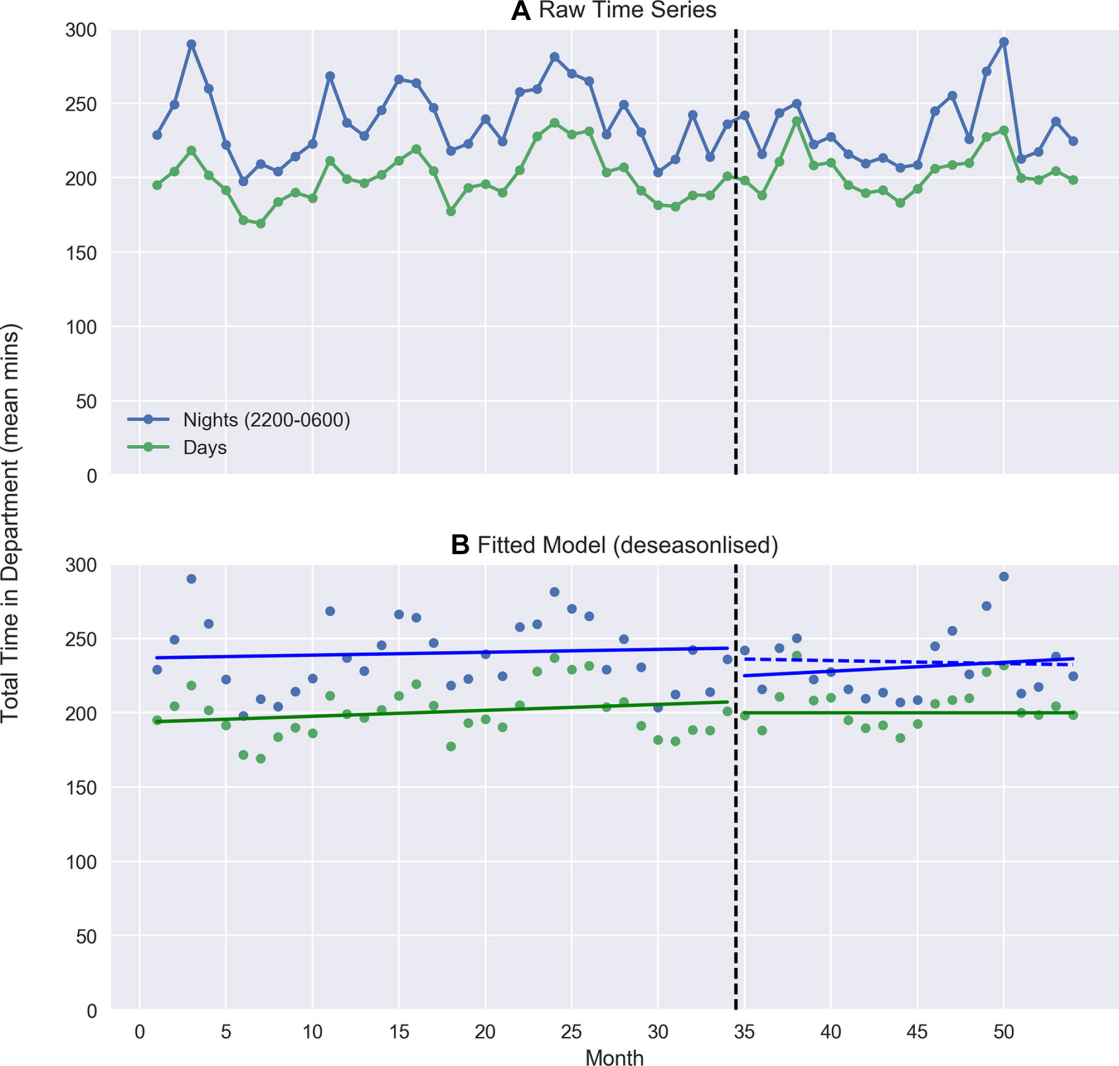
Effect of rostering consultants for overnight shifts on total time in department (deseasonalised trend). Raw day and nightime waiting times (A). Blue dashed line in (B) represents night-time counterfactual. The vertical black dashed line between months 34 and 35 represents the interruption in the time series.

Figure 1A,B illustrates the raw monthly time series and fitted ITS model for total time in department, respectively. Prior to the intervention, the average total time in department during the day (06:00–22:00) Monday to Thursday was 202 min (95% CI 197 to 206) with a small significant increase over time (a slope of 0.6; 95% CI 0.4 to 0.8 min/month, p<0.001). Average total time overnight was higher by 44 min (95% CI 38 to 50, p<0.001); with no substantive difference in trend from the day (−0.2; 95% CI −0.6 to 0.1, p=0.134). Following the introduction of a consultant overnight, there was no significant change in average time in department overnight relative to the day (−12 min; 95% CI −28 to 4, p=0.148) and no significant change in trend (0.5, 95% CI −0.6 to 1.6, p=0.382). The model was robust to a range of sensitivity analyses including adjustment for influential points in the winter of 2016/2017, monthly patient numbers and alternative autocorrelation structures. See the online supplementary appendix for full model output and details of sensitivity analyses.

#### Occupancy change

**Figure 2:**
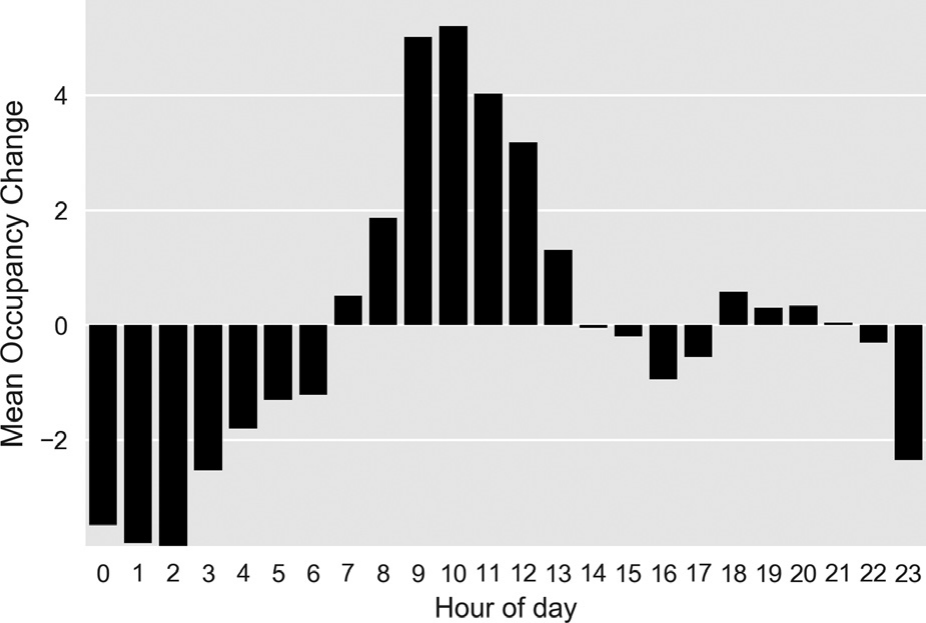
Occupancy change (arrivals minus departures) by hour of day.

Figure 2 breaks occupancy change into hourly averages. The department occupancy grows by an average of 3.0 patients per hour between 07:00 and 14:00 hours and remains relatively constant to 24:00 when occupancy begins to reduce by an average of 2.0 patients per hour.

#### Minutes to be first seen

**Figure 3:**
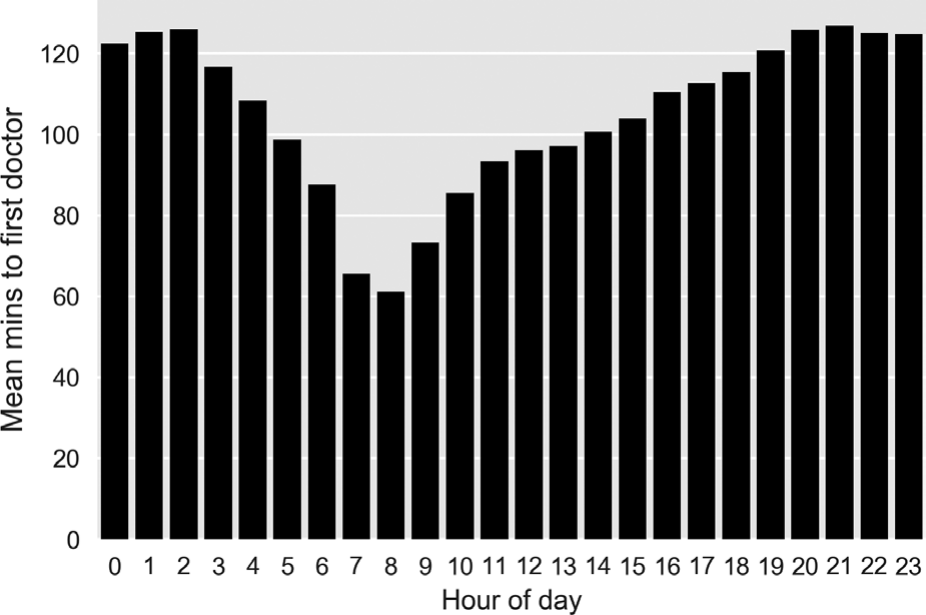
Average minutes to first doctor by time of day.

Figure 3 shows the average minutes for a patient to be first seen by a doctor across 24 hours. The minimum is 60 min, occurring at 08:00. The average time to be first seen grows from 08:00 to 19:00 hours where it levels off at an average of 120 min. A decline in average minutes to be seen begins at 03:00.

#### Adverse events

Between 24:00 and 08:00, across all days for the entire year, there was a total of three (0.9%) moderate and 0 severe AERs precluding further statistical testing.

### Qualitative results

Consultants reported working different lengths of shift, starting at 22:00 or 23:00 hours and finishing at 04:00 (a ‘casino’ shift), 06:00 or 07:00. Flexibility of hours worked enabled them to meet carer responsibilities, and/or to maintain well-being. Consultants had different practices, working as ‘clinician of the day’ and taking full responsibility for managing the department, or working as a clinical supervisor, directly seeing patients or a mixture of these, at night. Most of those interviewed said they did one to two nights consecutively.

Regardless of whether they currently worked at nights, consultant medical staff were sceptical about the advantages of consultant presence at night, and resistant to any further extension of the service to Friday to Sunday. Some noted that nights were opportunities to train and assess junior staff, skills and welcomed this, however several consultants suggested that juniors ‘need to experience running the floor’ (ie, being in charge) without a consultant present. In contrast, junior medical staff and the nurses interviewed held more mixed views, and suggested that having a senior decision maker to defer to could be useful as in the quote below.

When it is busy in the ED, the registrars can feel as though they are run ragged, even with a consultant, …with the consultant [there] at night they feel less stressed.

Consultant night working had an impact on their daytime presence because it was remunerated by additional days off. Additional night working, which would increase this, was identified by some consultants as a threat to team cohesion and communication. However, the greatest concern about night working centred on well-being and performance, particularly for older consultants, and those with young children. Interviewees said they ‘hated’ night shifts, that they felt ‘like a zombie’ especially between 03:00 and 05:00, and that their sleep patterns were disrupted/poor due to night work.

As I’ve got older I get more of the hangover feeling for the few days after a night shift. (Non-consultant)

By 4am you are a wreck. This has got worse as I got older, but especially now, with young children. (Consultant)

Two consultants stated that they did not feel particular ill effects from night working. But others reported problems, ranging from nausea to vomiting, dehydration and concerns about ability to drive safely after a night shift. Nurses and junior medical staff noted in the interviews that they had less favourable night working patterns than consultants.

Several consultants and all the non-consultant doctors felt that night working 7 days a week was likely to be introduced in future. Younger doctors, a few consultants and the nurses interviewed felt this might be beneficial for patient care, but all noted that long-term night working, for example, beyond the age of 55 years, was not ideal and might encourage senior medical staff to seek occupational health exemption from night working.

## Discussion

Our single-site study was unable to identify a clinically important impact of consultant night working on total time patients spend in the department. This result held after adjusting for seasonality, accounting for secular trends and a broad range of sensitivity analyses. Given the low frequency of severe adverse events, we were also unable to identify any meaningful improvements in AERs our proxy for safety.

In contrast to our results, the only other single-site studies from 2009 and 2010 report a positive effect on total time in department.^3 4^ It is notable that the waiting time performance of EDs in England has universally declined since these studies were completed.^2^ An explanation could be that other rate-limiting steps in processing patients (such as bed availability) have overtaken resourcing issues overnight. We also provide a larger retrospective sample of 54 months worked and use a stronger longitudinal design.

Our analysis suggests that if the prime motivation for overnight consultant working is improved waiting time metrics, the consultant workforce within our study site may be more usefully deployed at other times. Figures 2 and 3 illustrate how this ED struggles to manage the inflow during the day, with increasing occupancy (and presumed inefficiency), that is recovered overnight. Addressing this may improve night-time performance allowing night staff to manage current workload rather than attending to patients who have arrived hours earlier.

In contrast to our quantitative results, the views of staff at the study setting and responses from other similar EDs broadly agree with previous findings.^5 6^ These findings confirm fears about the negative impact of night working on staff well-being, performance and retention of medical staff. In the absence of compelling evidence about positive impacts on patient outcomes the perceived benefits of consultant presence at night, in supporting junior staff (and nurses), do not seem to outweigh these negatives.

The findings of our study should be interpreted with caution. First, although our study is the largest to date and uses a mixed methods design, it is a single-site analysis. Therefore, the findings particularly in respect to the quantitative intraday effects in waiting times cannot be generalised to other settings. Second, although we have attempted to account for time-varying confounders, such as patient numbers, and made use of daytime performance as a control in our analysis, it is possible that the consultants helped to mitigate against an increase in overnight waiting times. For example, hospitals in the region that did not introduce overnight working may have experienced increased overnight waiting times during the same period. Third, we focused on ‘waiting time’ metrics that are valued by the public, policymakers and hospital administrators. We cannot exclude the possibility that consultants make contributions to overnight working that are valued, but not measured in this study. For example, consultants may impact on the appropriateness of an admission, length of stay once admitted, efficient use of scarce hospital resources, unplanned reattendances, or reduction in patients who leave without being seen. Junior medical staff and nurses may feel more secure knowing that a consultant is present—as some interviewees suggested. Fourth, there may be performance-limiting steps outside of the EDs or a consultant’s control. For example, significant delays in emergency admissions due to high hospital occupancy.^10 11^ At our study site we found that the time between a decision to admit and admission was a consistent average of 2 hours across the day. We also note the CIs for our estimate of the potential reduction in total time in department include previous estimates of 20 min.^3^

In England, there is a push towards 24/7 consultant working. The benefits of such a move within an ED are unclear. Our study, limited by scope, was unable to find significant quality and safety benefits for patients. Before any widespread adoption of ED consultant night working, further evidence is required to address the uncertainty our study raises. At the minimum, EDs planning to switch to consultant grade doctor night working should prospectively evaluate the impact. Studies should consider a wide range of quantitative and qualitative outcomes for quality, safety and process as well as confounders; for example, measures of emergency admissions, benefits to trainee workforce and intrahospital rate-limiting effects such as delayed transfers of care or hospital occupancy. Given the complexity of the issue, such studies may need to be pragmatic and make use of ITS and non-equivalent controls. A gold standard approach would consider a larger scale multicentre randomised control trial and process evaluation. Initial consideration should be given to how interventions during normal working hours might mitigate the need for consultant presence at night.
