# Supplementary material for: A mixed methods study of the impact of consultant overnight working in an English Emergency Department
Source: Emerg Med J. 2018 Aug 9;36(5):298–302. doi: 10.1136/emermed-2018-207571 (PMC6580873; doi:10.1136/emermed-2018-207571)
Supplement: Supplementary data [file emermed-2018-207571supp001.docx]

**A mixed methods study of the impact of consultant overnight working in an English Emergency Department**

**Supplementary Appendix.**

Marion Penn^a^, Thomas Monks^a^, Catherine Pope^a^ and Mike Clancy^b^

^a^NIHR CLAHRC Wessex

University of Southampton

^b^Emergency Department, University Hospital Southampton FT

Southampton

# Supplementary Statistical Analysis

## Interrupted Time Series Analysis

An interrupted time series analysis using segmented regression [1] was used to analyse the effect of the intervention on overnight (10pm to 6am) performance. The data were divided into two segments: 1.) January 2013 to October 2015 and 2.) November 2015 to June 2017. We used daytime (6am to 10pm) performance as a quasi-control group in order to adjust for any intra-hospital effects that might influence ED such as improved ‘patient flow’ allowing more timely emergency admissions. To account for improved ED performance in the summer months (June to September) we introduced a dummy variable for summer to the regression equation. An exhaustive overview of Interrupted Time Series approaches can be found elsewhere [1-3].

In general the models took the form:

$Y_{jtk}=\beta_{o}+\beta_{1}.{time}_{t}+\beta_{2}\cdot{group}_{k}+ \beta_{3}\cdot{group}_{k}\cdot{time}_{t}+\beta_{4}\cdot{level}_{jt}+ \beta_{5}\cdot{trend}_{jt}+ \beta_{6}\cdot{{level}_{jt} \cdot group}_{k}+ \beta_{7}\cdot t{rend}_{jt}\cdot g{roup}_{k}+\varepsilon_{jtk}$

Where

- *Y_jtk_* is the predicted outcome of interest for intervention status j, at time *t*, for group *k*;
- β_0_ represents the baseline level for the control group;
- β_1_ is the pre-existing trend in the control group;
- β_2_ is the pre-existing difference in the level between the intervention and control groups;
- β_3_ is the pre-existing difference in trend between the intervention and control group;
- β_4_ is increase or decrease in the level of the control group;
- β_5_ is increase or decrease in the trend of the control group;
- β_6_ is the change in the level of the intervention group relative to the control group
- β_7_ is the change in the trend of the intervention group relative to the control group.
- ε_jtk_ is a normally distributed random error for status j at time t for group k. Errors may be correlated to errors from previous lags.

|  |
| --- |
| Figure S1: Illustrative example of ITS |

A key aspect of an ITS with a control time series is that the model assumes that what happened in the control series from pre to post would have happened in the intervention series if the intervention had not taken place. Figure S1 illustrates this concept for a hypothetical ITS evaluating the impact of an intervention on Total Time in an ED. The dashed blue line represents the *expected level and trend change* in the intervention series. This *counterfactual* line is the result of applying the observed level and trend change seen in the control series to the intervention series. The regression coefficients β_6_ and β_7_ are therefore interpreted as level and change changes *relative to the control group*. As a simple example, assume the level change in Total time the Department for the control group and intervention groups were estimated as 20 and 30 minutes respectively. The level change in the intervention relative to the control (β_6_) is a reduction of 10 minutes.

## ITS Procedure

All ITS analyses were conducted in R version 3.4.3 using the NLME (Non-linear mixed effects); car and tseries libraries. R code is available from the following git repository: <https://github.com/CLAHRCWessex/ed_consultant_ITS.git> . We followed the analysis procedure in [3]. Stationarity of the time series was confirmed using the augmented Dickey Fuller test. Autocorrelation of the error terms was identified through a combination a preliminary analysis of the residuals of an Ordinary Least Squares linear regression model using Durbin Watson statistics and ACF/PACF plots. ACF/PACF plots suggested an autoregressive process for error terms. Final modelling used Generalised Least Squares. Autocorrelation assumptions were tested for sensitivity using both alternative lags (up to an AR(16) as suggested by the PACF) and adding a moving average term. In all cases the substantive conclusions of the models were the same. Models were compared using Akaike information criterion (AIC). Normality of residuals from the final models were checked with Q-Q plots.

Influential points were assessed using Cook’s Distances (cut-off = 4/n , where n = 108). Sensitivity analyses adjusted for influential points. No data points were removed in the analysis.

In the final model of ‘total time in the department’ an adjustment for seasonality was incorporated using a single dummy variable for June through September. We tested the structural sensitivity of the model by adding eleven dummy variables to model each month separately. Only the variables for the summer months were significant (a reduction in average total time in department). The interaction between group (night versus day) and seasonality was not significant and was excluded from the final model. In all cases the conclusions from the model were the same. The final sensitivity analysis included an adjustment for the number of patients per month. Conclusions from the model were the same.

# Supplementary Quantitative Results

## Data filtering

There were 431,562 ED attendances in the full study period. We had access to the full ED database. We filtered the database for adult attendances (ages over 18) arriving Mon-Thu at all hours of the day. This left 186,500 attendances within our study population. This is further broken down in Table S1. All filtering was done using Python 3.6, Pandas 0.19.2 and numpy 1.11.3. A Jupyter notebook (‘pre-processing.ipynb’) containing all pre-processing code is available via <https://github.com/CLAHRCWessex/ed_consultant_ITS.git>

## Total Time in Department

Table S1 summarises the characteristics of the night and day time series.

| **Table S1: Period Characteristics** | | | | | |
| --- | --- | --- | --- | --- | --- |
| *Pre* | **N (%)** | **Pts/Mth (SD)** | **Age (IQR)** | **Age 65+** | **Ambulance (%)** |
| *Total* | 113,969 | 3,352 (246) | 47 (40.0) | 33,393 (29.3) | 52,044 (45.7) |
| Day | 87,189 (76.5) | 2,564 (190) | 48 (39.0) | 26,029 (29.9) | 36,409 (41.8) |
| Night | 26,780 (23.5) | 788 (70) | 44 (40.0) | 7,364 (27.5) | 15,635 (58.4) |
| *Post* |  |  |  |  |  |
| *Total* | 72,531 | 3,627 (278) | 47 (39.0) | 21,999 (30.3) | 32,778 (45.2) |
| Day | 56,206 (77.5) | 2,810 (226) | 48 (39.0) | 17,382 (30.9) | 23,613 (42) |
| Night | 16,325 (22.5) | 816 (69) | 44 (40.0) | 4,617 (28.3) | 9,165 (56.1) |

## ITS Results

Table S2 below details the model results for Total Time in Department. The model include a autoregressive term for residuals that was an AR(16). Sensitivity analysis considered down to AR(2) and ARMA models with an MA term up to 2. Both the time series and residuals were stationary. The coefficient for the summer season is β_8._

| Table S2: Final Model Results ~ ARMA(16,0) | | | | | | |
| --- | --- | --- | --- | --- | --- | --- |
|  | Coefficient | SE | t-value | *p* | 95% LCI | 95% UCI |
| β_0_ | 201.8 | 2.2 | 89.8 | 0.000 | 197.4 | 206.3 |
| β_1_ | 0.6 | 0.1 | 5.6 | 0.000 | 0.4 | 0.9 |
| β_2_ | 43.7 | 3.0 | 14.6 | 0.000 | 37.8 | 49.5 |
| β_3_ | -0.2 | 0.2 | -1.5 | 0.134 | -0.6 | 0.1 |
| β_4_ | -24.9 | 5.7 | -4.3 | 0.000 | -36.2 | -13.7 |
| β_5_ | 0.8 | 0.4 | 1.9 | 0.057 | 0.0 | 1.5 |
| β_6_ | -11.8 | 8.1 | -1.5 | 0.148 | -27.6 | 4.0 |
| β_7_ | 0.5 | 0.6 | 0.9 | 0.382 | -0.6 | 1.6 |
| β_8_ | -34.6 | 2.9 | -11.8 | 0.000 | -40.3 | -28.8 |

Model residual correlation structure: ARMA(16,0).

Coefficients.

| $\varphi_{1}$ | $\varphi_{2}$ | $\varphi_{3}$ | $\varphi_{4}$ | $\varphi_{5}$ | $\varphi_{6}$ | $\varphi_{7}$ | $\varphi_{8}$ |
| --- | --- | --- | --- | --- | --- | --- | --- |
| 0.010 | -0.299 | -0.159 | -0.073 | -0.079 | -0.271 | -0.110 | -0.834 |
| $\varphi_{9}$ | $\varphi_{10}$ | $\varphi_{11}$ | $\varphi_{12}$ | $\varphi_{13}$ | $\varphi_{14}$ | $\varphi_{15}$ | $\varphi_{16}$ |
| -0.254 | -0.346 | -0.215 | 0.130 | -0.281 | -0.207 | -0.111 | -0.374 |

## ITS Sensitivity to alternative residual modelling structures

Selection of the final model was done using the Akaike information criterion (AIC). Here was present an alternative simpler model AR(2). Substantive conclusions of both models is the same. The AR(16) model had a significantly lower AIC and smaller standard errors.

| Table S3: Final Model Results ~ ARMA(2,0) | | | | | | |
| --- | --- | --- | --- | --- | --- | --- |
|  | Coefficient | SE | t-value | *p* | 95% LCI | 95% UCI |
| β_0_ | 201.8 | 5.5 | 36.5 | 0.0 | 191.0 | 212.7 |
| β_1_ | 0.4 | 0.3 | 1.4 | 0.2 | -0.1 | 0.9 |
| β_2_ | 43.0 | 7.7 | 5.6 | 0.0 | 27.8 | 58.1 |
| β_3_ | -0.2 | 0.4 | -0.6 | 0.6 | -1.0 | 0.5 |
| β_4_ | -7.5 | 9.1 | -0.8 | 0.4 | -25.3 | 10.3 |
| β_5_ | 0.0 | 0.7 | 0.0 | 1.0 | -1.3 | 1.3 |
| β_6_ | -11.8 | 12.8 | -0.9 | 0.4 | -36.9 | 13.3 |
| β_7_ | 0.6 | 0.9 | 0.6 | 0.5 | -1.3 | 2.4 |
| β_8_ | -26.2 | 3.4 | -7.7 | 0.0 | -32.8 | -19.5 |

Model residual correlation structure: ARMA(2,0).

| $\varphi_{1}$ | $\varphi_{2}$ |
| --- | --- |
| 0.235 | -0.242 |

## ITS Sensitivity to influential points

Influential time points were found in winter of 2016/17 (Jan to Feb 2017; months 49 and 50 respectively). Higher average values for total time in department were expected in the winter months and the absolute values were consistent with winter peaks from previous years. The model was adjusted to test sensitivity to these points. In summary, conclusions remained the same. Quantitative results are presented in Table S4.

| Table S4: Adjusted model to account for wild points ~ ARMA(16,0) | | | | | | |
| --- | --- | --- | --- | --- | --- | --- |
|  | Coefficient | SE | t-value | *p* | 95% LCI | 95% UCI |
| β_0_ | 201.4 | 2.1 | 96.0 | 0.000 | 197.3 | 205.5 |
| β_1_ | 0.6 | 0.1 | 5.8 | 0.000 | 0.4 | 0.8 |
| β_2_ | 43.1 | 2.8 | 15.4 | 0.000 | 37.6 | 48.5 |
| β_3_ | -0.2 | 0.1 | -1.4 | 0.172 | -0.5 | 0.1 |
| β_4_ | -24.3 | 5.4 | -4.5 | 0.000 | -34.9 | -13.7 |
| β_5_ | 0.8 | 0.4 | 2.0 | 0.044 | 0.0 | 1.5 |
| β_6_ | -11.6 | 7.6 | -1.5 | 0.130 | -26.6 | 3.3 |
| β_7_ | 0.0 | 0.5 | 0.1 | 0.949 | -1.0 | 1.1 |
| β_8_ | -32.4 | 2.7 | -12.1 | 0.000 | -37.6 | -27.1 |
| β_9 (outlier)_ | 38.5 | 10.3 | 3.7 | 0.000 | 18.4 | 58.7 |

Coefficients.

| $\varphi_{1}$ | $\varphi_{2}$ | $\varphi_{3}$ | $\varphi_{4}$ | $\varphi_{5}$ | $\varphi_{6}$ | $\varphi_{7}$ | $\varphi_{8}$ |
| --- | --- | --- | --- | --- | --- | --- | --- |
| 0.016 | -0.260 | -0.208 | -0.114 | -0.100 | -0.296 | -0.125 | -0.193 |
| $\varphi_{9}$ | $\varphi_{10}$ | $\varphi_{11}$ | $\varphi_{12}$ | $\varphi_{13}$ | $\varphi_{14}$ | $\varphi_{15}$ | $\varphi_{16}$ |
| -0.236 | -0.330 | -0.217 | 0.073 | -0.291 | -0.283 | -0.125 | -0.334 |

**References**

1. Penfold, R.B. and F. Zhang, *Use of interrupted time series analysis in evaluating health care quality improvements.* Acad Pediatr, 2013. **13**(6 Suppl): p. S38-44 DOI: 10.1016/j.acap.2013.08.002.

2. Jandoc, R., A.M. Burden, M. Mamdani, L.E. Lévesque, and S.M. Cadarette, *Interrupted time series analysis in drug utilization research is increasing: systematic review and recommendations.* Journal of Clinical Epidemiology, 2015. **68**(8): p. 950-956 DOI: 10.1016/j.jclinepi.2014.12.018.

3. Wagner, A.K., S.B. Soumerai, F. Zhang, and D. Ross-Degnan, *Segmented regression analysis of interrupted time series studies in medication use research.* J Clin Pharm Ther, 2002. **27**(4): p. 299-309.
